# A proficient cost reduction framework for de-duplication of records in data integration

**DOI:** 10.1186/s12911-016-0280-9

**Published:** 2016-04-12

**Authors:** Asif Sohail, Muhammad Murtaza Yousaf

**Affiliations:** Punjab University College of Information Technology (PUCIT), University of the Punjab, Lahore, Pakistan

**Keywords:** Record linkage/de-duplication, Data integration, Record comparison reduction, Inverted index

## Abstract

**Background:**

Record de-duplication is a process of identifying the records referring to the same entity. It has a pivotal role in data mining applications, which involves the integration of multiple data sources and data cleansing. It has been a challenging task due to its computational complexity and variations in data representations across different data sources. Blocking and windowing are the commonly used methods for reducing the number of record comparisons during record de-duplication. Both blocking and windowing require tuning of a certain set of parameters, such as the choice of a particular variant of blocking or windowing, the selection of appropriate window size for different datasets etc.

**Methods:**

In this paper, we have proposed a framework that employs blocking and windowing techniques in succession, such that figuring out the parameters is not required. We have also evaluated the impact of different configurations on dirty and massively dirty datasets. To evaluate the proposed framework, experiments are performed using Febrl (Freely Extensible Biomedical Record Linkage).

**Results:**

The proposed framework is comprehensively evaluated using a variety of quality and complexity parameters such as reduction ratio, precision, recall etc. It is observed that the proposed framework significantly reduces the number of record comparisons.

**Conclusions:**

The selection of the linkage key is a critical performance factor for record linkage.

**Electronic supplementary material:**

The online version of this article (doi:10.1186/s12911-016-0280-9) contains supplementary material, which is available to authorized users.

## Background

With the introduction of corporate information systems and data warehouse, multiple data sources are linked and integrated together [1]. Due to this, the information systems get better by adding different dimensions to the information derived out of them. But at the same time, many superfluous records representing the same entity appear in the system, resulting in poor data quality. As more and more data are loaded and integrated into a data warehouse, the problems in data quality are multiplied (“garbage in, garbage out” - GIGO principle) [2]. The details about the impact of data quality problems on record linkage can be found in [3, 4].

Record linkage or de-duplication is used to eliminate superfluous or duplicate records. For a single dataset, the process of identifying the duplicate records is called de-duplication and for multiple datasets, the process is called record linkage [1]. This would be a trivial task if some unique identifier is available across different data sources to be linked. Unfortunately, this is a pathological scenario in the real world, especially in developing countries, where the patient’s record is typically accessed using some internal identifier. In such situations, linking has to be done on the basis of the attributes common to the data sources. The volume of data, variations in the data formats, data decay and noise in data are the major causes that resist the effective and efficient records linkage. Some other names used for record linkage are record matching, entity reconciliation, entity resolution [5, 6], object identification, duplication detection [7], data matching, or merge-purge problem [8].

### Applications

Record de-duplication or record linkage or has a pivotal role in data cleaning and data integration. The record linkage problem was formally introduced in 1969 [9] and has been getting massive attention in the current century due to the data explosion with the ubiquitous use of computers. The government and statistical agencies widely use it for census data, sample surveys, fraud detection, anti-terrorism etc.

In health sector, record linkage can be very effectively used for obtaining a comprehensive medical history of a patient. The complete information regarding a patient would normally be available at multiple hospitals or medical clinics that have been recorded over a period of time. For proper diagnosis and prescription, the unification of the scattered information through record linkage is of utmost importance. The record linkage has also proved to be very helpful in pharmaceutical research [10].

The business corporations use record de-duplication to improve customer relationship management and to save their mailing and printing cost. The same customer may be represented with different name variations or errors in other attributes. In the absences of record de-duplication, multiple copies of the same catalog may be sent to the same customer.

Web search engines use it for removing the duplicates before furnishing the query results to the user. Record de-duplication is of great advantage for de-duplicating citations in bibliographic databases. The identification of the duplicated citation is not a trivial task due to a variety of citation formats and spelling variations, e.g., “Jeffrey D. Ullman” vs. “Ullman, J.”. On web, open data is freely available to everyone. Using Linked Open Data (LOD), very interesting and useful data mining patterns can be explored [11]. Examples of LOD applications include linked data in libraries, linked data in biomedicine [10], linked government data etc.

### Record de-duplication process

**Fig. 1:**
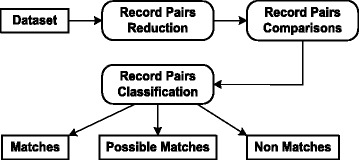
Record de-duplication process

A simple/naive approach for record de-duplication is to compare a record with every other record in the dataset. This approach would require *O*(*n*^2^) comparisons, for a dataset of ***n*** records., which is too high even for moderate size datasets. To manage the task economically, record de-duplication process shown in Fig. 1 is used. The process consists of the following phases:

1. Record pairs reduction
2. Record pairs comparisons
3. Record pairs classification

#### Record pairs reduction

For the reduction of record comparisons, inverted index is commonly used. It computes the indexing or hash key for each record and puts the records with hash key similar than a certain threshold in the same bucket, block, cluster, or pocket. Only the records residing in the same or neighboring buckets are compared in detail for classification and hence the number of record comparisons is reduced. Once the records have been bucketed, records to be compared with each other are selected using blocking or windowing method [7, 8, 12–20] discussed as under:

##### Blocking method

The standard blocking method makes record comparisons only among the records residing in the same block. The attribute(s) used for blocking form Blocking Key (BK) and its corresponding values are called Blocking Key Values (BKVs). The size and contents of blocks are dictated by the choice of BK. This makes the selection of BK an extremely important parameter towards the effectiveness and efficiency (reduction in number of record comparisons) of blocking. There is a tradeoff between small and large block size. An inverted index having small block sizes will make less record comparisons and may also miss out a significant number of true matches and vice-versa. There are some variants of blocking techniques with an explicit control over the block size [21–25].

Limitations of blocking include the placement of potential duplicates in different blocks and vice-versa. Moreover, blocks of significantly varying sizes are formed, when there is non-uniform or zipf distribution of blocking key values. To overcome these limitations, records may be placed in the same block using low threshold and/or multi-pass blocking may be used.

##### Windowing method

Windowing method extends the record comparisons to multiple adjacent blocks. It is also called Sorted Neighborhood Method (SNM) proposed by Herna’ndez and Stolfo [8] in mid 1990s. It uses sorted inverted index by sorting the records on the basis of sorting key values (SKVs). The records with the same SKV are grouped together in a common bucket. A fixed size window of size w (> 1) is then sled over the buckets and the record comparisons are made among all the record pairs falling in the same window. In this way, the records of adjacent blocks falling in the same window are also compared with each other which are not possible in blocking method [25].

The major challenge in using windowing technique is the selection of appropriate window size. There is a tradeoff between small and large window size. A small window size makes less record comparisons and may also miss out a significant number of true matches and vice-versa. In general, small window size works well for datasets with low to average number of duplicates and vice-versa. But the dilemma is that the dirtiness for the given real life datasets is unknown and hence the appropriate window size can only be discovered by trying different window sizes. One possible solution to overcome this problem is to use adaptive window size [15].

#### Record pairs comparisons

To compare two records, a set of attributes called linkage key is selected. The attributes are compared using different approximate comparison functions, such as, edit distance, q-gram distance, substring, soundex etc. [7, 12, 16, 26–29]. Assume that *Sim*(*a*, *b*) is a similarity function to compute similarity between two attributes *a* and *b*. The possible results of approximate comparison function would be:

- **Exactly Similar:***Sim*(*a*, *b*) = 1 (Agreeing value weight)
- **Completely Different:***Sim*(*a*, *b*) = 0 (Disagreeing value weight)
- **Partially Similar:** 0 < *Sim*(*a*, *b*) < 1 (Partial agreeing value weight)

The similarity scores of the selected fields are combined together and a vector called weight vector or comparison vector is formed, which is used for the follow-up classification phase.

#### Record pairs classification

The record pairs are classified as Match or Non-Match on the basis of the deterministic or probabilistic approach [3, 12, 14, 16, 23, 26, 27, 30, 31, 34] discussed as under:

##### Deterministic approach

A pair of records is classified as a match if and only if it completely agrees on all the attributes selected for linkage key. A slight variation in the stored values of the attributes will fail the exact match result. Since the values of linkage key may slightly differ from each other due to dirty data, therefore, the performance of deterministic approach will hugely depend upon the cleanness of the linkage key.

##### Probabilistic approach

Due to the data quality problems, an exact match between two records may not be possible even though they are referring the same object [28]. Consequently, it is better to make linkage decisions on the basis of approximate or probabilistic matching instead of exact matching. Two or more records deemed to be a match even if they have slight differences among them within some threshold value. The problem was named fuzzy duplicate elimination [37]. Let *F*(*r*_1_, *r*_2_) is a function used for the classification of record pairs. If *T*_*L*_ represents lower threshold and *T*_*U*_ represents upper threshold, then record pairs are classified as:

- **Match:***F* ≥ *T*_*U*_
- **Non-Match:***F* < *T*_*L*_
- **Possible Match:***T*_*L*_ ≤ *F* < *T*_*U*_

## Methods

**Fig. 2:**
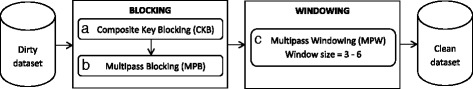
Proposed framework for record de-duplication

A framework shown in Fig. 2 is proposed to identify maximum duplicates with least number of record comparisons. The framework employs a hybrid technique using blocking phase followed by windowing phase.

The reason for choosing blocking and windowing methods in successions is that both blocking and windowing method have certain limitations (discussed earlier), when used alone. Blocking phase alone is bound to miss a significant number of duplicates for a dirty dataset containing huge number of duplicates. The errors in the blocking key places the potential duplicates in different blocks and hence such records are never compared with each other resulting in un-identified duplicates. Multipass blocking promises to identify more duplicates, but still a certain number of duplicates may remain uncovered due to massive dirtiness in the dataset. On the other hand, if the dirty dataset is directly input to the windowing method, then only a large window size (>15) can guarantee to identify maximum number of duplicates. Larger the window size, larger the number of matches found at the cost of huge number of record comparisons. Thus the windowing method alone is not cost effective for record de-duplication. To get around this situation, the proposed framework employs successive use of blocking followed by windowing method with small window size.

For building inverted index to be used in blocking and windowing, substring function is used to encode the indexing key because it is the least restrictive encoding function. Using substring function, potential duplicates will be placed in the same bucket even if their indexing keys agree at only the first few letters. Hence, there will be a great chance of correctly classifying the records after a detailed comparison based upon multiple fields.

### Blocking phase

The dirty dataset is input to the blocking phase first, where Composite Key Blocking (CKB) followed by Multipass Blocking (MPB) is used as shown in Fig. 2.

#### Composite key blocking (CKB)

It requires the least record comparisons because it is the most restrictive form of blocking. The records have to qualify composite condition in order to be placed in the same bucket. This will result in small block sizes and hence very small record comparisons.

##### Complexity analysis

Assuming a dataset of *n* records, each block will be assigned roughly $\frac{n}{b}$ records. Thus,

1 $$ \mathrm{Total}\ \mathrm{number}\ \mathrm{of}\ \mathrm{record}\ \mathrm{comparisons}=b\times \frac{\left[\frac{n}{b}\times \left(\frac{n}{b}-1\right)\right]}{2} $$

Suppose that K1 and K2 are the keys chosen for CKB. Let;

i = Number of distinct values for K1

j = Number of distinct values for K2

Let *i* ≥ *j*;

Using *Single Key Blocking – SKB* with K1 as blocking key;

$$ \mathrm{Number}\;\mathrm{of}\;\mathrm{blocks}=i\; of\; size\;1\;to\;n-i+1 $$

$$ \mathrm{Average}\;\mathrm{block}\;\mathrm{size}=\frac{n}{i} $$

2 $$ \mathrm{Total}\ \mathrm{number}\ \mathrm{of}\ \mathrm{record}\ \mathrm{comparisons}\left(\mathrm{on}\ \mathrm{average}\right)=i\times \frac{\left[\frac{n}{i}\times \left(\frac{n}{i}-1\right)\right]}{2} $$

Using *Composite Key Blocking – CKB* with both K1 and K2 as blocking key;

$$ \mathrm{Number}\;\mathrm{of}\;\mathrm{blocks}=i\ to\ ixj\; of\; size\;1\;to\;n-i+1 $$

$$ \mathrm{Average}\ \mathrm{number}\ \mathrm{of}\;\mathrm{blocks}=\frac{ixj}{2} $$

$$ \mathrm{Average}\ \mathrm{block}\ \mathrm{size}=\frac{2n}{ixj} $$

3 $$ \mathrm{Total}\ \mathrm{number}\ \mathrm{of}\ \mathrm{record}\ \mathrm{comparisons}\ \left(\mathrm{on}\ \mathrm{average}\right)=\frac{ixj}{2}\times \frac{\left[\frac{2n}{ixj}\times \left(\frac{2n}{ixj}-1\right)\right]}{2} $$

Now $\frac{n}{i}\ge \frac{2n}{ixj}$ as $1\ge \frac{2}{j}\ for\ j\ge 2$ and j is a whole number. Hence CKB will make lesser number of record comparisons as compared to SKB.

#### Multipass blocking (MPB)

It is used to overcome the placement of potential duplicates into different blocks due to dirtiness in the BKVs. If the potential duplicates are not placed in the same block using K1, then they get yet another chance to gather in the same block using K2. Thus, by increasing the number of passes, the probabilities of potential duplicates to gather in the same block increases. Nevertheless, multiple passes will also increase the number of record comparisons proportionally. Hence, the proposed framework uses two passes only. This is also due to the fact that blocking is not the only and terminal phase of the framework, rather it is to be followed by windowing phase. By the end of blocking phase, the input dirty dataset is reasonably de-duplicated and becomes appropriate for small sized windowing method.

##### Complexity analysis

Suppose that the first pass is performed using K1 and the second pass is performed using K2. Let;

i = Number of distinct values for K1

j = Number of distinct values for K2

4 $$ \mathrm{Total}\ \mathrm{number}\ \mathrm{of}\ \mathrm{record}\ \mathrm{comparisons}=i\times \frac{\left[\frac{n}{i}\times \left(\frac{n}{i}-1\right)\right]}{2}+j\times \frac{\left[\frac{n}{j}\times \left(\frac{n}{j}-1\right)\right]}{2} $$

### Windowing phase

After the completion of the blocking phase, the dataset is input to the windowing phase as shown in Fig. 2. This phase uses Multipass Windowing (MPW) discussed as under:

#### Multipass windowing (MPW)

For a massively dirty dataset, due to errors or noise in indexing keys, the potential duplicates are not likely to be placed in the buckets that are closer to each other. For this reason, massively dirty dataset requires larger window size as compared to a dataset with low to average dirtiness. Since the amount of dirtiness in the given dataset is not known in advance, hence the selection of appropriate window size remains a dilemma [35]. To get around this problem, MPW is used after the blocking phase. Two variations of blocking should transform the given dataset into relatively cleaner dataset and hence a small window size should be good enough to identify the residual duplicates that could not be identified using blocking.

##### Complexity analysis

Assuming a dataset of n records, each block will be assigned roughly $\frac{n}{b}$ records. Within a window of size *w*, total number of record identifiers will be $\frac{wn\ }{b}$.

$$ \mathrm{Total}\ \mathrm{number}\ \mathrm{of}\ \mathrm{record}\ \mathrm{pair}\ \mathrm{comparisons}\ \mathrm{in}\ \mathrm{the}\ \mathrm{first}\ \mathrm{window}\ \mathrm{position}=\frac{\frac{\mathrm{wn}}{\mathrm{b}}\left(\frac{\mathrm{wn}}{\mathrm{b}}-1\right)}{2} $$

For the remaining windows positions, one new inverted list of size $\frac{n}{b}$ is introduced leading to $\frac{\frac{\mathrm{n}}{\mathrm{b}}\left(\frac{\mathrm{n}}{\mathrm{b}}-1\right)}{2}$ comparisons. Along with that each record of (*w* − 1) inverted lists in the previous window will be compared with $\frac{n}{b}$ records of the newly introduced inverted list in the new window position. This requires $\left(\mathrm{w}-1\right)\frac{{\mathrm{n}}^2}{{\mathrm{b}}^2}$ comparisons. Hence, total number of record comparisons for windowing method is:

5 $$ =\frac{\frac{\mathrm{wn}}{\mathrm{b}}\left(\frac{\mathrm{wn}}{\mathrm{b}}-1\right)}{2}+\left(\mathrm{b}-\mathrm{w}\right)\left[\frac{\frac{\mathrm{n}}{\mathrm{b}}\left(\frac{\mathrm{n}}{\mathrm{b}}-1\right)}{2}+\left(\mathrm{w}-1\right)\frac{{\mathrm{n}}^2}{{\mathrm{b}}^2}\right] $$

For window of size 1, using w = 1 in the above equation will result in eqn. 1 derived for blocking method.

### Framework evaluation

**Fig. 3:**
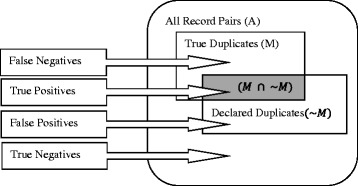
Possible results of duplicate detection

The possible classifications of the record pairs being evaluated by the framework is illustrated in Fig. 3 [25]. The quality of a record de-duplication process is accessed by the number of correctly reported matches and non-matches.

With synthesized datasets, for which, ground-truth or gold-standard data are available; the following analysis can be carried out [25]: Let;

- A = Set of all record pairs
- M = Set of true matching pairs
- ~*M* = Set of matching pairs reported by RL technique
- U = Set of true non-matching pairs i.e.
- ~*U* = Set of non-matching pairs reported by RL technique i.e. *A* – ~ *M*

Then, there can be the following possible outcomes of a record linkage process:

True Positives (TP) = Record pairs both in M and ~ *M* i.e. *M* ∩ ~ *M*

True Negatives (TN) = Record pairs both in U and ~ *U* i.e. *U* ∩ ~ *U*

False Positives (FP) = Record pairs that actually belongs to U but reported in ~ *M*

False Negatives (FN) = Record pairs that actually belongs to M but reported in ~ *U*

$$ M=TP+FN $$

$$ \sim M=TP+FP $$

$$ U=TN+FP $$

$$ \sim U=TN+FN $$

The above four possible outcomes of a record linkage process can be described using a confusion or error matrix [36] as shown in Table 1:

**Table 1:** Confusion Matrix

Actual	Classification by RL technique	
	Match ($\sim M$)	Non-match ($\sim U$)
Match (M)	True matches	False Non-matches
	True Positives (TP)	False Negatives (FN)
Non-match (U)	False Matches	True Non-matches
	False Positives (FP)	True Negatives (TN)

#### Calculating matching pairs of a dataset

Let;

n = Number of records in the dataset.

d = Number of duplicates per record.

$$ Duplicate\  Ratio(DR)=\frac{Number\  of\  duplicates}{n} $$

Then;

6 $$ \mathrm{No}.\ \mathrm{of}\ \mathrm{matching}\ \mathrm{pairs}\approx DR\times \frac{n}{d}\times \left[d+d\times \frac{d-1}{2}\right] $$

#### Quality and complexity parameters for evaluation

The record linkage techniques are assessed using the following quality and complexity parameters used in [4, 12, 13, 18, 19, 24, 26, 28, 32–35]:

$$ \begin{array}{l}\boldsymbol{Pairs}\ \boldsymbol{Q}\boldsymbol{uality}\ \left(\boldsymbol{P}\boldsymbol{Q}\right)\boldsymbol{or}\ \boldsymbol{Precision}=\frac{Matching\  pairs\  found}{Candiadate\  pairs\  generated\ }\\ {}\boldsymbol{Pairs}\ \boldsymbol{C}\boldsymbol{completeness}\ \left(\boldsymbol{P}\boldsymbol{C}\right)\boldsymbol{or}\ \boldsymbol{R}\boldsymbol{ecall}=\frac{Matching\  pairs\  found}{Total\  matching\  pairs}\\ {}\boldsymbol{R}\boldsymbol{eduction}\ \boldsymbol{R}\boldsymbol{atio}\ \left(\boldsymbol{R}\boldsymbol{R}\right)=1-\frac{Candidate\  pairs\  generated}{Total\  record\  pairs\kern0.5em }\\ {}\boldsymbol{F}-\boldsymbol{Score}=1-\frac{2\times PC\times RR}{PC+RR\kern0.5em }\end{array} $$

#### Datasets for evaluation

A framework can be evaluated either using public datasets or synthesized datasets. A limitation of public datasets is that true match status of the record pairs may not be available. An alternate is to use synthesized data sets, for which, ground-truth or gold-standard data is available. The framework is evaluated using the synthetic datasets, whose metadata is given in Table 2. The datasets have been generated using database generator (DBGen) utility of Febrl and are publically available with Febrl [20]. The datasets are populated with artificial entries using probabilistic data generation for de-duplication. These datasets have been used in previous research [17, 24, 26].

**Table 2:** Datasets for FRAMEWORK Evaluation

Dataset name	No. of fields	No. of records	No. of original records	No. of duplicate records
Dataset-A	12	1000	500	500
Dataset-C	12	1000	600	400

The fields for dataset-A and dataset-C are given as under:

*given_name, surname, street_number, address_1, address_2, suburb, postcode, state, date_of_birth, age, phone_number, soc_sec_id*

In dataset-A, there is one duplicate against an original record, one modification per duplicate record and maximum one attribute is modified in the duplicate record. In dataset-C, there can be up to nine duplicates against an original record, maximum ten modifications per duplicate record and maximum three modifications per attribute.

## Results and discussion

For experimental evaluation, Febrl (Freely Extensible Biomedical Record Linkage) system is used [20]. The experiments are carried out using the permutations given in the Table 3.

**Table 3:** Permutations for Experimental Evaluation

Indexing technique	Methodology	Encoding function for indexing key	Field comparison functions
1. Blocking	• Single Key Blocking (SKB) • Composite Key Blocking (CKB) • Multipass Blocking (MPB)	• Soundex (SDX) • Substring-4 (SB4) • Substring-3 (SB3)	• Soundex • Edit-Distance • Q-gram
2. Windowing with window sizes 3, 6, 9, …, 30	• Single Key Windowing (SKW) • Composite Key Windowing (CKW) • Multipass Windowing (MPW)		

The impact of the following variations on the results is analyzed in the experiments:

- Indexing key(s)
- Encoding function used for indexing key(s)
- Single pass vs. multiple passes of a technique
- Window size on the number of record comparisons and on the quality of data matching process (for windowing only)
- The performance of techniques using dirty dataset (dataset-A) and massively dirty dataset (dataset-C)

For all the experiments, the fields used for comparisons (also called linking fields) and comparison functions are given in Table 4. The selected fields have less than 5 % missing values and hence are appropriate for detailed record level comparisons. To set a benchmark for the experiments to be carried out in the following sub-sections, initial experiments are carried out using full index approach. This approach makes all the possible record comparisons and hence can identify the maximum number of matches. The results of the experiments using full index are presented in Table 5, which shows that the number of matches is less than 0.2 % of the number of record comparisons. Precision using full index approach is very small (0.000993 and 0.002210 for dataset-A and dataset-C respectively). This concludes that full index approach is prohibitively expensive for very large datasets.

**Table 4:** Linking Fields and Comparison Functions

Linking fields	Comparison function
postcode	Edit Distance
address_1	Q-gram
soc_sec_id	Edit Distance
given_name	Soundex/Substring
surname	Soundex/Substring

**Table 5:** Results of Experiment using Full Index

Dataset	Dataset-A	Dataset-C
Record Comparisons	499500	499500
Classified Matches	496	1054
Classified possible matches	2	135
Pairs Quality or Precision	0.000993	0.002210

### Blocking experiments

Blocking experiments are categorized into 3 sets i.e., Single Key Blocking (SKB), Composite Key Blocking (CKB) and Multipass Blocking (MPB). CKB and MPB are proposed in the framework and SKB is used to compare its results with the blocking categories proposed in the framework. All the experiments are carried out for dataset-A (DA) as well as for dataset-C (DC). The fields selected for blocking key(s) has/have less than 3 % missing values. The setup for each experiment set is given in Table 6.

**Table 6:** Setup for BLOCKING Experiments (X = A OR C)

Experiment category	Exp. code	Blocking key	Encoding function for blocking key
Single Key Blocking (SKB)	DX-SKB	*given_name*	1. Soundex (SDX) 2. Substring4 (SB4) 3. Substring3 (SB3)
Composite Key Blocking (CKB)	DX-CKB	*given_name* + *surname*	
Multipass Blocking (MPB)	DX-MPB	*given_name* (Pass1), *surname* (Pass2)	

#### Results & discussion

The results of all the experiments performed using dataset-A and dataset-C are presented in Tables 7 and 8 respectively. The best value for each of SKB, CKB and MPB is written in **bold** face and the worst value is written in *italic*.

**Table 7:** Results of blocking methods for dataset-A

Blocking method	Single key blocking (SKB)			Composite key blocking (CKB)			Multipass blocking (MPB)		
Blocking Keys	SDX	SB4	SB3	SDX	SB4	SB3	SDX	SB4	SB3
Record Comparisons	**3562**	4096	*11080*	**455**	484	*631*	**4242**	5279	*17191*
Matches	*454*	474	**482**	*442*	476	**486**	**496**	*494*	495
F-Score	*0.949*	0.969	**0.971**	*0.938*	0.975	**0.985**	**0.992**	0.989	*0.978*

**Table 8:** Results of blocking methods for dataset-C

Blocking method	Single key blocking (SKB)			Composite blocking key (CKB)			Multipass blocking (MPB)		
Blocking keys	**SDX**	**SB4**	**SB3**	**SDX**	**SB4**	**SB3**	**SDX**	**SB4**	**SB3**
Record comparisons	**3639**	4175	*10678*	**249**	583	*986*	**4348**	5340	*15652*
Matches	*542*	848	**956**	*231*	551	**755**	*719*	1008	**1030**
F-Score	*0.675*	0.886	**0.939**	*0.358*	0.684	**0.831**	*0.805*	**0.970**	**0.970**

The following observations can be made on the basis of experimental results presented in Tables 8 and 9:

- As discussed in proposed framework (section II), the least restrictive blocking key (such as SB3) identified the highest number of matches at the expense of additional record comparisons.
- CKB made least number of record comparisons and still it identified an excellent number of matches. This is due to small block sizes formed by CKB as discussed in section II.
- MPB identified the highest number of matches at the expense of additional record comparisons.
- MPB is less sensitive towards the choice of encoding function used for blocking key. As can be seen from Table 7, the number of matches of MPB remains almost the same irrespective of the choice of encoding function used for blocking key. However, unlike dataset-A, the number of matches for MPB are not totally immune to the choice of encoding function used for blocking key. As can be seen from Table 8, the number of matches is greater for truncated keys (SB4 and SB3) than SDX.
- The performance of MPB is marginally better using SB3 than using SB4, whereas, the performance of both SKB and CKB is significantly better when used with SB3.

**Table 9:** Setup for WINDOWING Experiments (X = A OR C)

Experiment category	Exp. code	Description	Sorting key
Single key windowing (SKW	DX-SKW-SDX	Dataset X - Single Key Windowing - Soundex encoding	*given_name*
	DX-SKW-SB4	Dataset X - Single Key Windowing - Substring4 encoding	
Composite key windowing (CKW)	DX-CKW-SDX	Dataset X - Composite Key Windowing - Soundex encoding	*given_name* + *surname*
	DX-CKW-SB4	Dataset X - Composite Key Windowing - Substring4 encoding	
Multipass windowing (MPW)	DX-MPW-SDX	Dataset X - Multipass Windowing - Soundex encoding	*given_name*, *surname*
	DX-MPW-SB4	Dataset X - Multipass Windowing - Substring4 encoding	

### Windowing experiments

The setup for each of the windowing experiments is presented in Table 9. For each variant, the experiments are performed using 10 different window sizes, i.e., 3, 6,…,30.

#### Results & discussion

The results of windowing experiments are presented in Table 10. For brevity, only the results for window sizes 3, 6, 12, 21, and 30 for dataset-A and dataset-C are presented in Table 10. For each dataset, Table 10 is divided into three partitions on the basis of widowing method used such SKW, CKW and MPW. Each partition is further subdivided into two on the basis the encoding function used for sorting key such as Soundex (SDX) and Substring4 (SB4). The best value of F-Score within each sub-partition is written in bold face and the worst value within each sub-partition is written in *italic*.

**Table 10:** Results of windowing variants (Dataset-A and Dataset-C)

Dataset	Dataset-A					Dataset-C				
Window size	3	6	12	21	30	3	6	12	21	30
Record Pairs : SKW-SDX	9551	17151	32679	54322	75768	8476	14777	27629	47081	65030
Matches : SKW-SDX	469	478	481	485	486	764	864	918	965	978
F-Score : SKW-SDX	0.959	**0.961**	0.948	0.929	*0.906*	*0.832*	0.886	0.904	**0.908**	0.895
Record Pairs : SKW-SB4	10271	18591	34882	58769	81591	9808	16380	30259	50809	70322
Matches : SKW-SB4	479	482	483	484	484	900	949	967	979	981
F-Score : SKW-SB4	**0.969**	0.963	0.948	0.923	*0.898*	0.910	**0.930**	0.926	0.911	*0.891*
Record Paris : CKW-SDX	3539	7186	14437	24966	35314	3342	6624	13033	22469	31713
Matches : CKW-SDX	469	477	482	488	490	519	662	783	862	912
F-Score : CKW-SDX	0.965	**0.970**	0.968	0.963	*0.954*	*0.656*	0.765	0.840	0.878	**0.897**
Record Paris : CKW-SB4	3651	7430	14884	25905	36655	3803	7409	14240	24426	34359
Matches : CKW-SB4	487	488	491	491	492	750	863	922	955	969
F-Score : CKW-SB4	**0.983**	0.981	0.976	0.965	*0.954*	*0.826*	0.892	0.918	**0.925**	0.923
Record Paris : MPW-SDX	13858	25968	49722	82615	114045	12158	21982	41884	70643	96960
Matches : MPW-SDX	496	496	496	496	496	889	976	1015	1032	1034
F-Score : MPW-SDX	**0.982**	0.970	0.944	0.907	*0.868*	0.902	**0.938**	0.936	0.912	*0.883*
Record Paris : MPW-SB4	15614	29191	54977	91679	125521	14434	25208	46783	78219	107261
Matches : MPW-SB4	494	494	494	494	494	1022	1031	1032	1034	1035
F-Score : MPW-SB4	**0.978**	0.964	0.936	0.894	*0.852*	**0.968**	0.961	0.939	0.905	*0.870*

The experimental results are evaluated on the basis of the number of records comparisons, number of matches and F-Score discussed as under:

##### Number of record comparisons

**Fig. 4:**
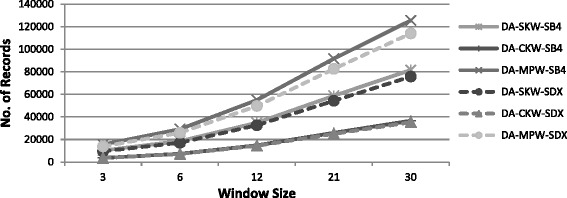
Number of record comparisons of windowing variants using SDX (Dataset-A)

As discussed in section II and can be seen in Fig. 4, the number of record comparisons increases as we increase the window size. The number of record comparisons also depend upon the choice of encoding function used for sorting key (SDX or SB4). For a given window size, less restrictive key, such as, SB4 makes more record comparisons than SDX. Since MPW operates in multiple passes, therefore, its rate of increase in the number of record comparisons is the highest, whereas the rate of increase in the number of record comparisons is the lowest with CKW (as per claim and justification given in the proposed framework).

##### Number of matches

The number of matches and its rate of increase depend both upon three categories of windowing method (SKW, CKW and MPW) and the encoding function used for sorting keys (SDX or SB4). It can be seen from Table 10 that SB4 identified more number of matches than SDX for a given window size. This is again due to less restrictive characteristics of SB4 as discussed earlier. Using SB4, larger buckets are formed and hence the potential duplicates are more likely to be placed in a small window.

**Fig. 5:**
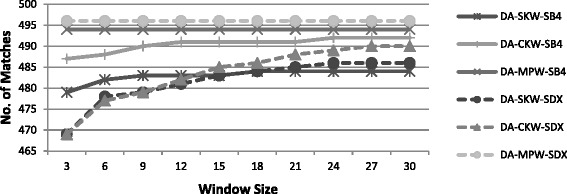
Number of matches of windowing variants using SDX (Dataset-A)

In Fig. 5, the number of identified matches of SKW, CKW and MPW using SDX and SB4 are plotted for varying window for dataset-A. The number of matches remained constant with MPW and increased both with SKW and CKW as the window size was increased. As can be seen from Table 10, for dataset-C, using MPW-SB4, the increase in the number of matches with increasing window sizes is very nominal. This concludes that small window size such as three is appropriate for MPW as the larger window size doesn’t yield much benefit.

It can be seen from Table 10 that for dataset-A, CKW-SB4 identified the similar or even higher number of matches by making far lesser record comparisons than SKW at the corresponding window sizes. Using window of size 15, CKW made 17990 record comparisons and identified 485 matches in contrast to 39814 record comparisons and 483 matches of SKW. Thus, this combination can be very effective for record de-duplication of huge dataset provided that the dataset is not massively dirty.

##### F-score

**Fig. 6:**
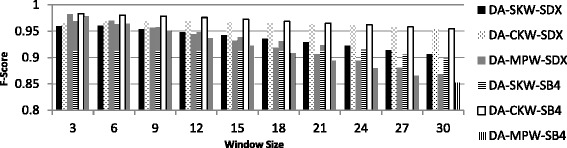
F-Score of windowing variants using SDX and SB4 (Dataset-A)

F-Score represents a trade-off between the number of record comparisons and the number of matches. As can be seen from Table 10, a window of certain size cannot be fixed to achieve the highest F-Score for all the windowing variants. For dataset-C, F-score initially increased for both SKW and CKW, and then it started decreasing for a window of size greater than nine for SKW and for a window size of greater than 24 for CKW. The results highlight the challenge in the selection of an appropriate window size. In Fig. 6, F-Score of SKW, CKW and MPW using SDX and SB4 are plotted for varying window sizes for dataset-A. The figure shows that, for all windowing variants, F-Score is the highest with small window sizes (3–6) both for SDX and SB4. Later on it decreases with larger window sizes. The rate of decrease in F-score is maximum with MPW and minimum with CKW both for SDX and SB4.

#### Conclusions of windowing phase

In all the windowing experiments, it was observed that different window sizes were required to get the best results under different windowing variants and encoding functions used for keys. The window sizes that offered the best results are summarized in Table 11. From the Table, it can be seen that, due to the amount of dirtiness, dataset-C required larger window sizes as compared to dataset-C. Since, it is difficult to work out the amount of dirtiness in any real life dataset, therefore, appropriate window size is difficult to suggest. The proposed framework resolves this issue by eliminating the need of selecting window size.

**Table 11:** Best window sizes under for dataset-A and dataset-C

Windowing variant	Window size for Dataset-A		Window size for Dataset-C	
	SDX	SB4	SDX	SB4
Multipass Windowing – MPW (Highest matches)	3–6	3–6	21–24	6–9
Composite Key Windowing – CKW (Least comparisons)	21–24	6–9	30	30
Single Key Windowing - SKW	21–24	6–9	30	21–24

### Comparison of blocking and windowing

While comparing blocking phase with windowing phase, it is quite obvious that blocking makes lesser record comparisons and may identify lesser duplicates than windowing with window size > 1. However, multi-pass blocking may identify similar number of duplicates that are identified by windowing. To evaluate this, a performance comparison between multi-pass blocking and windowing using dataset A and dataset C is made on the basis of the results presented in Tables 7, 8 and 10.

**Fig. 7:**
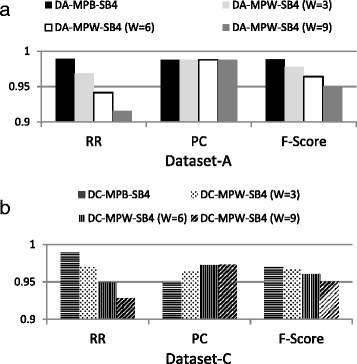
Comparison of MPB and MPW for Dataset-A (**a**) and Dataset-C (**b**)

Figure 7 plots the quality parameters RR, PC and F-score of MPB and MPW with window sizes 3–9 for dataset-A and dataset-C. It can be seen in Fig. 7a that for dataset-A, the number of matches (PC) is same both for MPB and different window sizes of MPW. However, blocking made lesser record comparisons and offered better F-Score than windowing. Thus, MPB proved to be a better method for dataset-A. Figure 7b shows that for dataset-C, even with small window of size three, windowing has better PC than blocking. This is mainly due to the massive dirtiness of dataset-C. It infers that for massive dirty dataset, the use of windowing method cannot be ruled out in order to identify the maximum number of matches.

To de-duplicate a massively dirty dataset (like dataset-C), an extreme approach can be to use MPW with large widow size, say, 30. Such an approach made 107261 record comparisons and identified 1035 matches. Using the proposed framework, only 20321 record comparisons were made to identify the same number of matches as shown in Table 12. Thus, the proposed framework reduces the number of record comparisons by a factor of 5.28.

**Table 12:** Number of comparisons using proposed framework

Phases of the proposed framework	Number of comparisons
CKB using SB4	583
MPB using SB4	5304
MPW using SB4	14434
Total	20321

The above discussion concludes that we cannot pick a single method between blocking and windowing that can work well for both dataset-A and dataset-C. Thus to get the best results, both methods should be used in succession as proposed in the framework. While using windowing method, the uncertainty in the selection of appropriate window size can be handled using the proposed framework. With the proposed framework, once the data has been cleansed using CKB and then by MPB, a small size window (3–6) should be a safe option. This is also evident from the results of dataset A, where a window size of three proved to be sufficient enough to catch the maximum duplicates.

### Future work

We plan to carry out experiments on larger datasets and evaluate their results for the proposed framework. It is also planned to perform the experiments using different thresholds and analyze their impacts on the results. The impact of composite key comprising of more than two fields can be investigated as well. Multipass blocking and windowing has a huge potential for parallel computing, so an attempt can be made in this direction. Similarly, the scalability issue of a given technique is another avenue of further research.

The decision of selecting the threshold for records pair classification greatly depends upon the choice of comparison functions and the quality of the underlying data. For example, a study conducted in [16] concludes that q-gram comparison function returns the highest comparison value and Jaro’s algorithm returns the lowest comparison value, when two partially similar strings are compared. So, an appropriate threshold range can be proposed for different comparison functions. Also, the suitability of a comparison functions for a given type/quality of data can be investigated. Similarly, the development of multilingual phonetic encoding functions is another avenue of further research.

In recent years, the issue of privacy preserving record linkage has been investigated. There is a great room to amend the current state of the art record linkage techniques to ensure privacy preserving.

## Conclusions

On the basis of the experimental results and discussion, the following conclusions are drawn:

- The successive use of blocking and windowing increased the number of identified duplicates.
- The proposed framework reduced the number of record comparisons significantly.
- The proposed framework eliminates the need of trying different window sizes for different datasets and requires a small window of size 3–6 irrespective of the amount of dirtiness in a dataset.
- The number of record comparisons (and hence the number of identified duplicates) increased as the indexing key was made less and less restrictive.
- Composite Key Blocking (CKB) makes the least number of record comparisons.

### Ethics approval and consent to participate

Not Applicable.

### Consent for publication

Not Applicable.

### Availability of data and materials

The datasets used in the manuscript are available as **additional files** named dataset_A_1000 and dataset_C_1000 (Additional files 1 and 2) [20]. These files contain the synthetic data used in this paper. The files are in comma separated, delimited format and is viewable in Microsoft Excel or any text editor.

## Additional files

Additional file 1: Dataset-A with one duplicate against an original record and one modification per duplicate record. (CSV 92 kb) Additional file 2: Dataset-C with up to 9 duplicates against an original record. (CSV 90 kb)

## Abbreviations

BK: blocking key
SKB: single key blocking
CKB: composite key blocking
MPB: multipass blocking
SKW: single key windowing
CKW: composite key windowing
MPW: multipass windowing
SDX: soundex
SB4: substring-4
SB3: substring-3
RR: reduction ratio
PC: pairs completeness

## References

[1] Bleiholder J, Naumann F (2008). Data fusion. ACM Comput Surv.

[2] Rahm E, Do HH (2000). Data cleaning: problems and current approaches. IEEE Data Engineering Bulletin.

[3] Herzog TN, Scheuren FJ, Winkler WE. Data quality and record linkage techniques. Springer Science & Business Media; 2007.

[4] Randall SM (2013). The effect of data cleaning on record linkage quality. BMC Med Inform Decis Mak.

[5] Whang SE, Garcia-Molina H (2010). Entity resolution with evolving rules. PVLDB.

[6] Whang SE, Garcia-Molina H (2011). Developments in generic entity resolution. IEEE Data Engineering Bulletin.

[7] Elmagarmid AK, Ipeirotis PG, Verykios VS (2007). Duplicate record detection: a survey. IEEE Trans Knowl Data Eng.

[8] Hernandez MA, Stolfo SJ (1995). The merge/purge problem for large databases.

[9] Fellegi IP, Sunter AB (1969). A theory for record linkage. J Am Stat Assoc.

[10] Samwald M (2011). Linked open drug data for pharmaceutical research and development. J Cheminform.

[11] Bauer F, Kaltenböck M (2011). Linked open data: the essentials.

[12] Christen P, Goiser K. Quality and complexity measures for data linkage and deduplication. In: F. Guillet, H. Hamilton (eds). Quality Measures in Data Mining, Studies in Computational Intelligence, vol. 43. Springer; 2007, pp. 127–151.

[13] Christen P. A survey of indexing techniques for scalable record linkage and deduplication. Knowledge and Data Engineering, IEEE Transactions on 24.9. 2012. p. 1537–55.

[14] Christen P. Data Matching, Concepts and Techniques of Record Linkage, Entity Resolution, and Duplicate Detection. Springer; 2012.

[15] Draisbach, U., Naumann, F., Szott, S., & Wonneberg, O. (2012, April). Adaptive windows for duplicate detection. In Data Engineering (ICDE), 2012 IEEE 28th International Conference on (pp. 1073-1083). IEEE.

[16] Elfeky MG, Verykios VS, Elmagarmid AK (2002). TAILOR: a record linkage toolbox.

[17] Goiser K, Christen P (2006). ‘Australasian Data Mining Conference’ (AusDM’06), vol. 61.

[18] Gu L, Baxter R, Vickers D, C. Rainsford C (2003). Record linkage: current practice and future directions.

[19] Patrick L, Fankhauser P, Min Tjoa A, Trujillo J (2005). A Precise Blocking Method for Record Linkage. DaWaK 2005, LNCS 3589.

[20] Christen P (2008). Febrl: an open source data cleaning, deduplication and record linkage system with a graphical user interface.

[21] Michelson M, Knoblock CA (2006). Learning blocking schemes for record linkage.

[22] Giang P (2014). A machine learning approach to create blocking criteria for record linkage. Health Care Manag Sci.

[23] Data Integration Manual (2006). Statistics New Zealand.

[24] Gu L, Baxter R (2004). Adaptive filtering for efficient record linkage.

[25] Naumann F, Herschel M. An introduction to duplicate detection. Synthesis Lectures on Data Management 2.1. 2010. 1–87.

[26] Gu L, Baxter R (2006). Decision models for record linkage. Selected Papers from AusDM, Springer LNCS 3755.

[27] Maggi F (2008). A Survey of Probabilistic Record Matching Models, Techniques and Tools.

[28] Christen P (2006). A comparison of personal name matching: Techniques and practical issues. Workshop on Mining Complex Data, held at IEEE ICDM. Hong Kong.

[29] Odell M, Russell R (1918). The soundex coding system. US Patents 1261167.

[30] Baxter R, Christen P, Churches T (2003). “A comparison of fast blocking methods for record linkage.” ACM SIGKDD. Vol. 3.

[31] Köpcke H, Rahm E (2010). Frameworks for entity matching: a comparison. Data Knowl Eng.

[32] Draisbach U, Naumann F (2009). A comparison and generalization of blocking and windowing algorithms for duplicate detection. Workshop on Quality in Databases, held at VLDB. Lyon.

[33] Fawcett T (2004). ROC graphs: notes and practical considerations for researchers. Mach Learn.

[34] Jiang L (2009). Measuring and Comparing Effectiveness of Data Quality Techniques.

[35] Yan S, Lee D, Kan MY, Giles LC (2007). Adaptive sorted neighborhood methods for efficient record linkage. ACM/IEEE-CS joint conference on Digital Libraries.

[36] Han J, Kamber M, Pei J. Data mining: concepts and techniques. Elsevier; 2011.

[37] Chaudhuri S, Ganti V, Motwani R (2005). Robust identification of fuzzy duplicates.

